# C_4_-like *Sesuvium sesuvioides* (Aizoaceae) exhibits CAM in cotyledons and putative C_4_-like + CAM metabolism in adult leaves as revealed by transcriptome analysis

**DOI:** 10.1186/s12864-024-10553-2

**Published:** 2024-07-13

**Authors:** Christian Siadjeu, Gudrun Kadereit

**Affiliations:** 1 Prinzessin Therese von Bayern Lehrstuhl für Systematik, Biodiversität & Evolution der Pflanzen, Ludwig-Maximilans-Universität München, Menzinger Str. 67, Munich, 80638 Germany; 2 Botanischer Garten München-Nymphenburg Und Botanische Staatssammlung München, Staatliche Naturwissenschaftliche Sammlungen Bayerns, Menzinger Str. 65, Munich, 80638 Germany

**Keywords:** *Sesuvioideae*, *S. sesuvioides*, Drought stress, C_4_-like, CAM, RNA-seq, Transcriptome, C_4_-like-CAM

## Abstract

**Background:**

The co-occurrence of C_4_ and CAM photosynthesis in a single species seems to be unusual and rare. This is likely due to the difficulty in effectively co-regulating both pathways. Here, we conducted a comparative transcriptomic analysis of leaves and cotyledons of the C_4_-like species *Sesuvium sesuvioides* (Aizoaceae) using RNA-seq.

**Results:**

When compared to cotyledons, phosphoenolpyruvate carboxylase 4 (*PEPC4*) and some key C_4_ genes were found to be up-regulated in leaves. During the day, the expression of NADP-dependent malic enzyme (*NADP-ME*) was significantly higher in cotyledons than in leaves. The titratable acidity confirmed higher acidity in the morning than in the previous evening indicating the induction of weak CAM in cotyledons by environmental conditions. Comparison of the leaves of *S. sesuvioides* (C_4_-like) and *S. portulacastrum* (C_3_) revealed that *PEPC1* was significantly higher in *S. sesuvioides*, while *PEPC3* and *PEPC4* were up-regulated in *S. portulacastrum*. Finally, potential key regulatory elements involved in the C_4_-like and CAM pathways were identified.

**Conclusions:**

These findings provide a new species in which C_4_-like and CAM co-occur and raise the question if this phenomenon is indeed so rare or just hard to detect and probably more common in succulent C_4_ lineages.

**Supplementary Information:**

The online version contains supplementary material available at 10.1186/s12864-024-10553-2.

## Introduction

In a number of eudicot families, C_4_ photosynthesis evolved in ancestrally succulent lineages (see [1], for an overview), prominent examples are Chenopodiaceae [2], Aizoaceae-Sesuvioideae [3], Portulacaceae [4] and Zygophyllaceae [5]. Some families like Aizoaceae and Portulacaceae also use crassulacean acid metabolism (CAM) as carbon concentrating mechanism (CCM). Both CCMs share the same core metabolic enzymes, both evolved repeatedly multiple times, however, it seems that they rarely co-occur. So far, the co-occurrence of C_4_ and CAM was verified for only four genera, *Portulaca* (Portulacaceae; [6]), *Spinifex* (Poaceae; [7]), *Ottelia* (Hydrocharitaceae; [8]) and *Trianthema* (Aizoaceae; [9]). Recent evidence demonstrated that C_4_ and CAM are operating in the same cells in *Portulaca oleracea* under drought conditions [10]. This integration is plausible due to several copies of core C_4_ genes [i.e. *phosphoenolpyruvate carboxylase* (*PEPC*)] that are recruited for C_4_ and CAM, respectively, sharing a set of biochemical reactions. Detecting the co-occurrence of C_4_ and CAM is laborious, requires living collections and an experimental approach, which is why this phenomenon has not been documented very often. However, we hypothesize that it might be more common in succulent C_4_ lineages than currently known.

C_4_ photosynthesis is an adaptive evolutionary response to the harmful effect of photorespiration under hot and dry growing conditions, by concentrating CO_2_ around RUBISCO [11]. This allows for a remarkably efficient photosynthesis, as well as water and nitrogen use. The C_4_ pathway is a complex combination of anatomical and biochemical specialization. In succulent C_4_ lineages the C_4_ anatomy is particularly diverse [1, 12]. Often the Kranz cells are not arranged as an inner wreath around the vascular bundles like in plants with typical Kranz anatomy, but form a continuous inner chlorenchyma layer around the central water storage tissue of the leaf (e.g., [3, 13]). The general pathway in which CO_2_ is converted to bicarbonate (HCO_3_^−^) by carbonic anhydrase in mesophyll cells (MCs) and then fixed to the 3-carbon molecule phospho*enol*pyruvate by the enzyme PEPC to form the 4-carbon molecule oxaloacetate (OAA) unifies all plants with C_4_ photosynthesis. OAA is then either reduced to malate or transaminated to aspartate. After diffusing to an adjacent Kranz cell, malate or aspartate is primarily decarboxylated by the enzymes either NADP-dependent malic enzyme (NADP-ME) or NAD-dependent malic enzyme (NAD-ME). This decarboxylation releases CO_2_ in high concentrations around RUBISCO and ensures high photosynthetic efficiency. This carbon concentration mechanism (CCM) is supported and facilitated by a decrease in the ratio of mesophyll to Kranz cells as opposed to the C_3_ ancestors.

Unlike C_4_ photosynthesis, the CCM of CAM photosynthesis is temporally asynchronous in a single-cell system. During the night, plants open stomata, and CO_2_ is fixed and converted to malate, which is stored in the vacuole as malic acid. During the day, stomata are closed and stored malate is transported out the vacuole and decarboxylated to release CO_2_ that is then fixed by RUBISCO and enters the Calvin cycle for sugar production. This asynchronous carbon fixation system allows plants to keep their stomata closed to avoid water loss through evapotranspiration during the hottest period of the day. Thus, plants with this type of metabolism are able to grow in hot and dry environments. While it is straight forward to detect obligate CAM plants by means of a strong carbon isotope signal and consistent differences between morning and evening acid concentrations, it is laborious to detect facultative or weak CAM plants that only induce CAM under stress [14, 15]. Weak CAM can neither be detected by carbon isotope ratios in C_3_ species nor in C_4_ species. In C_3_ species, the discrimination of RUBISCO towards the heavier C isotope and in C_4_ species the much higher activity of the PEPC in the C_4_ pathway hides the low CAM signal.

Aizoaceae comprise annual or perennial herbs, rarely shrubs or trees, growing in tropical and subtropical regions, predominantly in South Africa [16]. Most species of the family are succulent and many, especially from subfamily *Mesembryanthemoideae* and *Ruschioideae* are documented CAM plants [14]. In Aizoaceae, C_4_ photosynthesis is restricted to subfamily Sesuvioideae and likely evolved multiple times [3]. A striking diversity of leaf anatomical types and the occurrence of both biochemical subtypes of C_4_ (NAD-ME and NADP-ME) can be observed. In addition to this photosynthetic diversity, two species from Sesuvioideae have been reported to activate low CAM under drought, i.e., the C_3_ species *Sesuvium portulacastrum* and the C_4_ species *Trianthema portulacastrum* ([9, 15]). Yet another species of this subfamily arouses curiosity: *Sesuvium sesuvioides*, a succulent C_4_-like species with uncommon C_4_ features and photosynthetic plasticity during leaf aging. Structural, physiological, and biochemical analysis of *Sesuvium sesuvioides* indicated a relatively high MC/bundle sheath cell (BSC) ratio and the presence of RUBISCO large subunit together with PEPC in the MCs [3]. Furthermore, a decrease of C_4_ enzyme activities was observed from young to mature to senescent leaves [17]. Although Bohley et al. [17] did not observe any CAM activity under well-watered conditions, they did not exclude the existence of CAM under dry conditions.

Such species that exhibit photosynthetic variability may contain footprints left from the evolution of CAM and C_4_ photosynthesis and thus provide useful information to either disentangle or gain new insight into the evolution and regulation of C_4_ and CAM metabolism. From this perspective, the photosynthetic flexibility in subfamily *Sesuvioideae* represents an excellent potential study group. We need such models as with climate change, many agricultural regions approaching their potential peak of productivity [18], and with an estimated population of 10 billion people by 2050, C_4_ and CAM represent a promising way to increase productivity and hence yield to meet global demands for food owing to their intrinsic ability to thrive in hot and dry environments. Thus, both CCMs are targets for genetic engineering into C_3_ species. Substantial efforts taken in the past to introduce C_4_ and CAM features into C_3_ plants failed to reach the envisioned goals due to lack of knowledge of C_4_ and CAM photosynthesis at the system level [19]. Therefore, mechanisms underlying C_4_ and CAM anatomical structure, gene-specific expression, and regulation network in C_4_ must be clarified further [19], and each new mosaic stone will help to solve the conundrum.

To test our hypothesis that *S. sesuvioides* operates combined C_4_ and CAM photosynthesis, we (1) performed a comparative transcriptome analysis between cotyledons and young leaves of *S. sesuvioides* (C_4_-like species) and young leaves of *Sesuvium portulacastrum* (C_3_ species) under stressful conditions: high light intensity and drought. We then (2) investigated the integration of C_4_-like + CAM in *S. sesuvioides* via the identification of candidate genes linked to C_4_ and CAM previously identified in *Portulaca* [20]. Finally, we explored the regulatory elements controlling C_4_-like and C_3_ pathways. Our analyses revealed that *S. sesuvioides* is operating weak CAM in cotyledons and C_4_-like + CAM in leaves as proved by gene expression analysis and supported by acid titration. Moreover, C_4_-like + CAM candidate genes were found up-regulated during the day suggesting the integration of C_4_-like + CAM metabolism in *S. sesuvioides*.

## Materials and methods

### Plant materials

Plants of *S. sesuvioides* (C_4_-like) and *S. portulacastrum* (C_3_) were grown from seeds and cuttings respectively, in the experimental greenhouse of the Munich-Nymphenburg Botanical Garden, Germany. *Sesuvium sesuvioides* seeds were collected from a location situated ~ 80 km east of Sendelingsdrif, Karas, Namibia [~ 28.20946°S, 17.28936°E, 208 m altitude, voucher: Klak 2431 (BOL)] and *S. portulacastrum* plant materials were collected in Texas, USA (MSB Serial number 0394523; year collected: 2007). For simplicity, we will sometimes use C_4_ instead of C_4_-like. Germinated seedlings (about 1 cm) and one-year-old plants of *S. sesuvioides* and one-year-old plants of *S. portulacastrum* were transferred to climate chambers with the following parameters: photoperiod light/dark 14 h/10 h, 60% humidity, [CO_2_] = 400 ppm, maximum light intensity = 785 μmol/m^2^/s, day/night temperature of 25/22 °C. Plants were watered every 2 days. This created stressful conditions specifically drought with changes in leaves colors as shown in the pictures (Additional file 1). Samples for transcriptome were harvested two weeks after transferring the plants to the climate chamber. Three young adult leaves of three plants of each species were collected during the day (1 pm) and for *S. sesuvioides* cotyledons three replicates of four plants were harvested during day (at 1 pm) and night (9 pm, one hour after the light was off). The plant *S. sesuvioides* was first identified by Cornelia Klak and later confirmed by Katharina Bohley. *Sesuvium portulacastrum* was also identified by Katharina Bohley. Vouchers of *S. sesuvioides* (LS 257) and *S. portulacastrum* (LS 221) have been deposited at the Herbarium MSB with serial numbers M-0356568 and M-0356569 respectively.

### Titratable acidity

Possibility of CAM activity under stressful conditions was investigated in cotyledons and leaves of *S. sesuvioides* and leaves of *S. portulacastrum* via comparative titratable acidity between 30 min before the end and 30 min before the beginning of the light period (19.30 h and 5.30 h, respectively). Cotyledons and leaves were harvested and snap-frozen in liquid nitrogen and stored at -20 °C. Since cotyledons were small, eight plants of *S. sesuvioides* for each harvesting time were collected. Stored cotyledons and leaves were chopped and weighed. About 50 mg of the cotyledons and leaves material were incubated at 60 °C in 20% ethanol for 60 min. The extract obtained was aliquoted into three replicates of the same volume (1 mL). The extracted acid was neutralized by adding 0.01 M NaOH in 1ul increments [21].

### RNA extraction and sequencing

Total RNA was extracted from leaves and cotyledons as described by Siadjeu et al. [22] using innuPREP Plant RNA Kit (Analytik Jena AG, Jena, Germany). Total RNA quality control was performed using the 2100 Bioanalyzer (Agilent Technologies) and Agarose gel electrophoresis. Messenger RNA was purified from total RNA using poly-T oligo-attached magnetic beads. After quality control and fragmentation, the first-strand cDNA was synthesized using random hexamer primers followed by the second-strand cDNA synthesis. The library was ready after end repair, A-tailing, adapter ligation, size selection, amplification, and purification.

### Transcriptome analysis

**Fig. 1:**
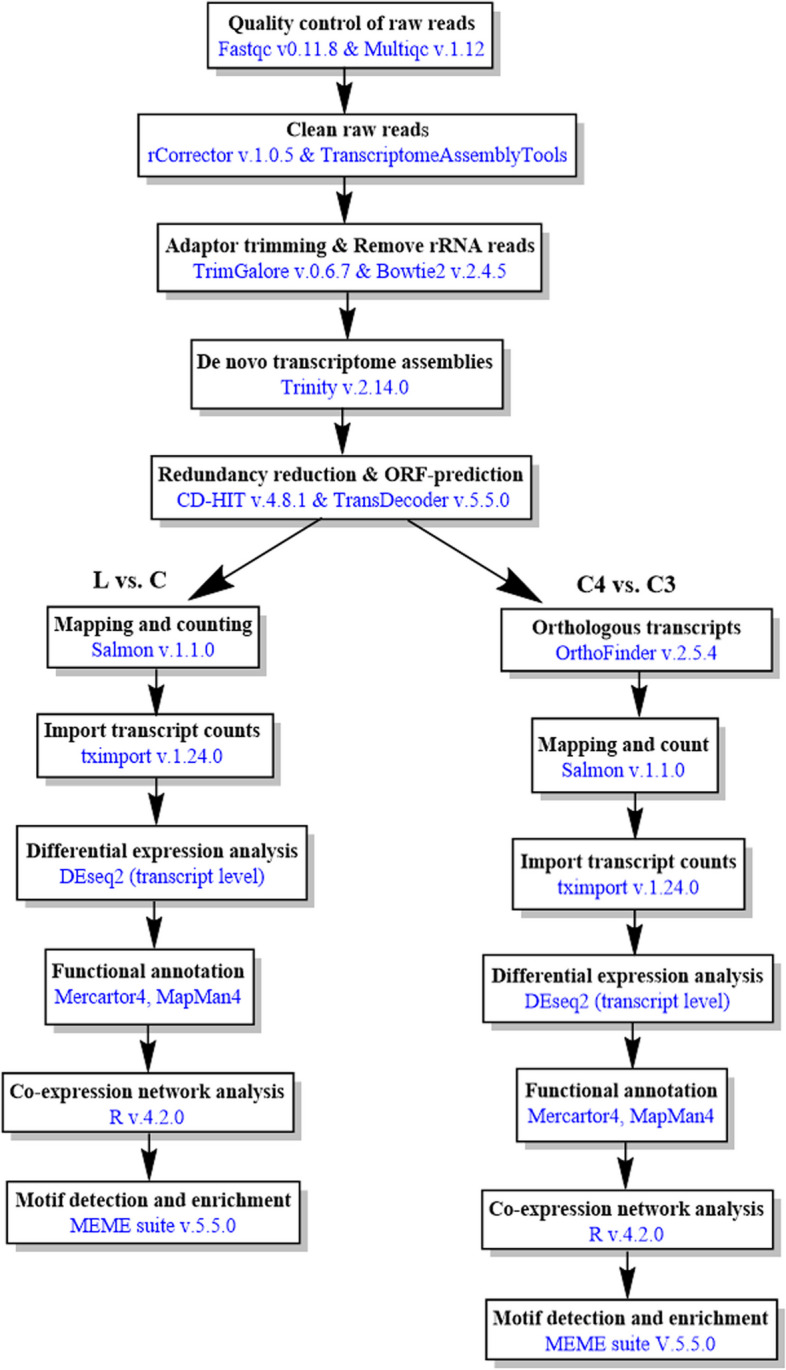
Workflow of transcriptome data analysis. The blue color represents the software used. L: leaves, C: cotelydons

An overview of the bioinformatics pipeline presenting software and respective versions used is presented in Fig. 1. Sequence read quality control was assessed using FastQC (https://www.bioinformatics.babraham.ac.uk/projects/fastqc/) and summarized with MultiQC [23]. Random sequencing errors in reads were corrected with a k-mer-based method implemented in Rcorrector [24] and uncorrectable reads were removed from the reads using TranscriptomeAssemblyTools (Fig. 1) (https://github.com/harvardinformatics/TranscriptomeAssemblyTools). Low-quality reads and adapters were filtered using TrimGalore v0.6.7 (https://github.com/FelixKrueger/TrimGalore/releases). The rRNA reads were removed by aligning trimmed reads against the SILVA v-138 rRNA database using Bowtie2 v.2.4.5 [25]. De novo transcriptome assembly was performed using Trinity v.2.14.0 [26] with the following parameters (Trinity –seqType fq –SS_lib_type RF –max_memory 200G –min_contig_length 300 –CPU 16). The de novo transcriptome assembly quality was first confirmed by aligning cleaned reads back to the corresponding de novo transcriptome assembly using Bowtie2 v.2.4.5. Secondly, the BUSCO score (odb10), which evaluates the completeness of the transcriptome, was determined using BUSCO v4 [27]. For the downstream analysis, the initial assembly of each species was processed as follows. We reduced the transcriptome data by clustering transcripts with 98% similarity with CD-HIT v4.8.1 [28]. Then, we selected only transcripts harboring coding sequences with TransDecoder v.5.5.0 (https://github.com/sghignone/TransDecoder). TransDecoder performs a precomputed blastX alignment to the Uniprot protein sequence database to improve the prediction of open reading frames. Finally, we used the indexes of transcripts containing coding sequences to subset the initial assemblies. The reduced transcriptomes were used for differential expression analysis. For differential expression analysis, we exclusively focused on *S. sesuvioides* when comparing leaves collected during the day to cotyledons collected both during the day and at night. For the comparison between C_3_ and C_4_-like species, we used young adult leaves collected during the day.

### Transcript quantification and differential expression analysis

We quantified transcript abundance with Salmon by aligning the reads of each species to its reduced transcriptome (Fig. 1). The software tximport v. 1.24.0 [29] was used to import transcript level abundances, estimated counts, and effective lengths for differential expression analysis. For comparison between the leaves of C_3_ and C_4_-like species, we searched for orthologs between their reduced transcriptomes using OrthoFinder v2.5.4 [30]. Based on the orthology between C_3_ and C_4_-like species, we obtained unique indexes by blasting C_3_ and C_4_-like species transcripts against each other. The best blast hits with the lowest e-value and high bit scores were selected and considered homologous. Transcripts of the C_4_-like species were used as a reference for the unique ID. If a C_4_-like species transcript was hit multiple times with transcripts of the C_3_ species, only one randomly selected transcript was kept to get a similar number of transcripts for differential expression analysis. Finally, the unique C_4_-like ID was changed in the Salmon output of the C_3_ species before importing the data with tximport (Script is available at https://github.com/Siadjeu/Sesuvioideae_C4-CAM). We assessed differential expression with the program Deseq2 [31]. Transcripts with p-value and p-adjusted as false discovery rate < 0.05 were considered significantly expressed andLog2FC was set > 1.

### Pathway and gene ontology (GO) annotation

Metabolic pathways and annotations of differentially expressed (DE) transcripts were assigned via the tool Mercator4 [32]. Swiss-prot protein sequences database and prot-scriber were included to improve the annotations. Unassigned transcripts were manually assigned based on the knowledge of the molecular functions in C_4_/CAM, photorespiration, and starch metabolism. We assigned GO terms to DETs using Blast2GO through OmicsBox with cutoff = 55, GO weight = 5, e-value = 1.e-5, HSP-hit coverage cutoff = 80 and hit filter = 500. We enriched the GO terms using Fisher’s exact test via OmicsBox.

### Phylogenetic analysis of PEPC isoforms

To investigate whether various isoforms of *PEPCs* are involved in CAM or C_4_ photosynthesis, we conducted a phylogenetic analysis of *PEPC* derived from our study alongside three distinct genes (*PPC-1E1*, *PPC-1E2* and *PPC-2*) that encode PEPC from 35 different species. It has been found that *PPC-1E1* is consistently involved in both CAM and C_4_ photosynthesis [20]. We obtained the protein sequences of these genes from the UniProt database (https://www.uniprot.org/, accessed on 06.03.2024). To generate proteins from the transcript sequence of *PEPC* discovered in our study, we employed TransDecoder v5.7.1 (https://github.com/TransDecoder/TransDecoder), and extracted the longest open reading frames of these transcripts. We constructed a multiple sequence alignment using MAFFT v.7.520 [33], and subsequently built an unrooted maximum likelihood tree using RAxML v8.2.13 [34].

### Co-expression network analysis

The co-expression analysis was performed using the unsupervised machine learning algorithm k-means in R. We used the three most popular methods for determining the optimal cluster: the Elbow and silhouette [35] methods and gap statistic [36]. The normalized read counts of DETs were used. The maximum number of clusters (k) was set to 10. If transcription factors (TFs) and phytohormones were clustered with C_4_-like or CAM genes, they were considered candidate TFs and phytohormones controlling C_4_-like or CAM photosynthesis. The k-means clustering script is available underhttps://github.com/Siadjeu/Sesuvioideae_C4-CAM.

### Motif detection and enrichment

Transcript sequences of k-means clusters were analyzed for motif identification and enrichment. The program MEME suite v.5.5.0 [37] was employed to detect de novo motifs with the following parameters: E-value threshold = 0.05, minimum motif size = 6 bp. We checked for motif redundancy with TOMTOM [38] using the motif database JASPAR nonredundant core 2022. We enriched the detected motifs using AME [39] with the following parameters:ame --verbose 1 --oc. --scoring avg --method fisher --hit-lo-fraction 0.25 --evalue-report-threshold 0.05 --control --shuffle-- --kmer 2 MemeUpC_3_vsC_4_photoStach.fasta motif_db/JASPAR.

## Results

### Titratable acidity

**Fig. 2:**
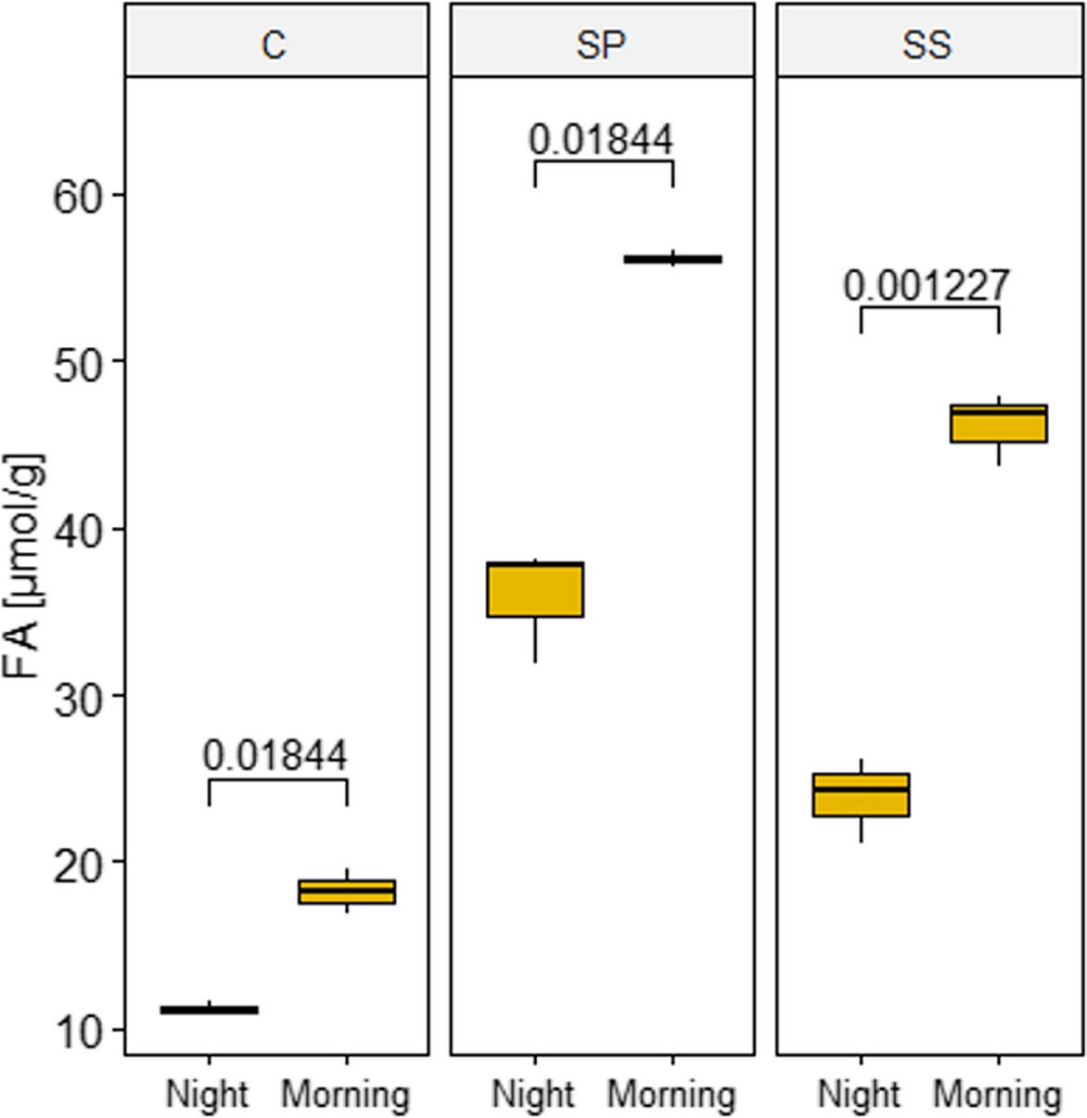
Boxplots of morning-night differences in titratable acidity of cotyledons (C) and leaves (SS) of *S. sesuvioides* and leaves of *S. portulacastrum* (SP). Values in the graph indicate the adjusted *p* values of significant differences between morning and night. FA: refers to free acidity, which is measured based on a normalization against the fresh weight of a sample

To investigate CAM induction in leaves and cotyledons of *S. sesuvioides* and leaves of *S. portulacastrum*, we conducted titratable acidity tests. Interestingly, the tests revealed significant overnight acid accumulation in leaves (t-test, adjusted *p*-value = 0.001) and cotyledons (t-test, adjusted *p*-value = 0.018) of *S. sesuvioides* and in leaves of *S. portulacastrum* (t-test, adjusted *p*-value = 0.018) (Fig. 2). Variations in nocturnal acidification were about 7 μmol/g fresh weight of free acids (FA) in cotyledons of *S. sesuvioides*, and 22 and 20 μmol/g FA in leaves of *S. sesuvioides* and *S. portulacastrum*, respectively. Similar overnight acid accumulation has been reported in the leaves of *S. portulacastrum* under drought stress [40]. Moreover, acid accumulations of 15 and 18 ∆H^+^ have been observed in drought stress in *Portulaca amilis* and *P. oleracea*, respectively, which induce CAM photosynthesis under stress conditions [41]. These differences in acidity between the morning and night indicate a weak CAM induction. Therefore, we analyzed the transcriptome profiles of *S. sesuvioides* and *S. portulacastrum* in relation to C_4_ and CAM photosynthesis.

### Transcriptome assembly and quality assessment

The initial transcriptome assemblies of *S. sesuvioides* and *S. portulacastrum* contained 313,669 and 248,314 transcripts that were highly complete and little fragmented (C:96.2%, F:2.1) and (C:95.9 F:2.4), respectively (Table 1). Only 27 % of transcripts (85,170) of *S. sesuvioides* and 31 % of *S. portulacastrum* (76,264) were predicted to possess coding sequences. However, no significant changes were observed in the BUSCO scores (*S. sesuvioides*: C: 95.3 %, F: 2.5 %; *S. portulacastrum*: C: 95.1 %, F: 2.8 %). Transcripts with coding sequences were used for differential expression analysis between cotyledons and leaves of *S. sesuvioides*. For cross-species differential expression analysis, 43,769 and 40,429 orthologous transcripts were identified in *S. sesuvioides* (C: 84.9 %, F: 3.5 %) and *S. portulacastrum* (C: 82.8 %, F: 4.6 %) with an overall alignment of 73 % and 76 %, respectively.

**Table 1:** Statistics of assembly and alignment for transcriptomes of *Sesuvium sesuvioides* and *S. portulacastrum*

Species	Software	Number of config	% of reads alignment	Complete BUSCO (%)	Fragmented BUSCO (%)
*Sesuvium sesuvioides* (C_4_-like)	Trinity	313669	98.79	96.2	2.1
	CD-HIT	232335	98.76	95.5	2.5
	TransDecoder	85170	93.25	95.3	2.5
	OrthoFinder	43769	73.05	84.9	3.5
*Sesuvium portulacastrum* (C_3_)	Trinity	248314	98.62	95.9	2.4
	CD-HIT	195534	98.57	95.4	2.8
	TransDecoder	76264	90.65	95.1	2.8
	OrthoFinder	40429	75.7	82.8	4.6

### Differential expression analysis across *Sesuvium* species

**Fig. 3:**
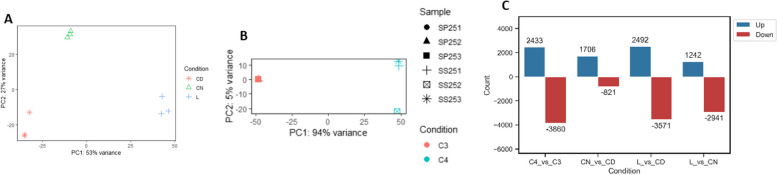
Transcriptome expression patterns of *Sesuvium*. **A** Principal component analysis (PCA) of leaves vs cotyledons of *S. sesuvioides*, CD: cotyledons day, CN cotyledons night, L: leaves. **B** PCA of *S. sesuvioides* vs *S. portulacastrum*, SP251-253: *S. portulacastrum* replicates 1:3, SS251: *S. sesuvioides* replicates 1:3. **C** Number of differential expressed transcripts: C4_vs_C3, between leaves of *S. sesuvioides* (C_4_-like) and *S. portulacastrum* (C_3_); CN_vs_CD, between cotelydons of *S. sesuvioides* collected during the night and during the day; L_vs_CD, between leaves and cotelydons of *S. sesuvioides* both collected during the day; L_vs_CD, between leaves and cotyledons *S. sesuvioides* both collected during the night. The dash (-) sign indicates that transcripts are down-regulated

Two comparative analyses were carried out: (1) between leaves and cotyledons of *S. sesuvioides*, and (2) between leaves of *S. sesuvioides* and leaves of *S. portulacastrum*. Leaves and cotyledons of *S. sesuvioides* were clearly separated based on their expression profile (Fig. 3A). Likewise, C_3_ and C_4_-like species were clustered according to their photosynthetic type mainly along PC1 (Fig. 3B). A total of 6,063 transcripts were found to be significantly DE between leaves and cotyledons of *S. sesuvioides* during the day (L and CD), of which 2,492 were up-regulated in leaves and 3,571 in cotyledons (Fig. 3C). When comparing leaves during the day (L) and cotyledons of *S. sesuvioides* during the night (CN), 1242 were up-regulated in leaves and 2,941 in cotyledons. Comparison of cotyledons between night (CN) and day (CD) revealed that 1,706 and 821 transcripts were found to be up-regulated at night and day, respectively. Between the C_3_ (*S. portulacastrum*) and C_4_ (*S. sesuvioides*) species, we found 20,867 orthologous transcripts in a 1:1 relationship. Out of these orthologs, 3,860 transcripts were significantly up-regulated in the C_3_ species and 2,433 in the C_4_-like species (Fig. 3C).

### Functional annotation of DETs between *S. sesuvioides and S. portulacastrum*

**Fig. 4:**
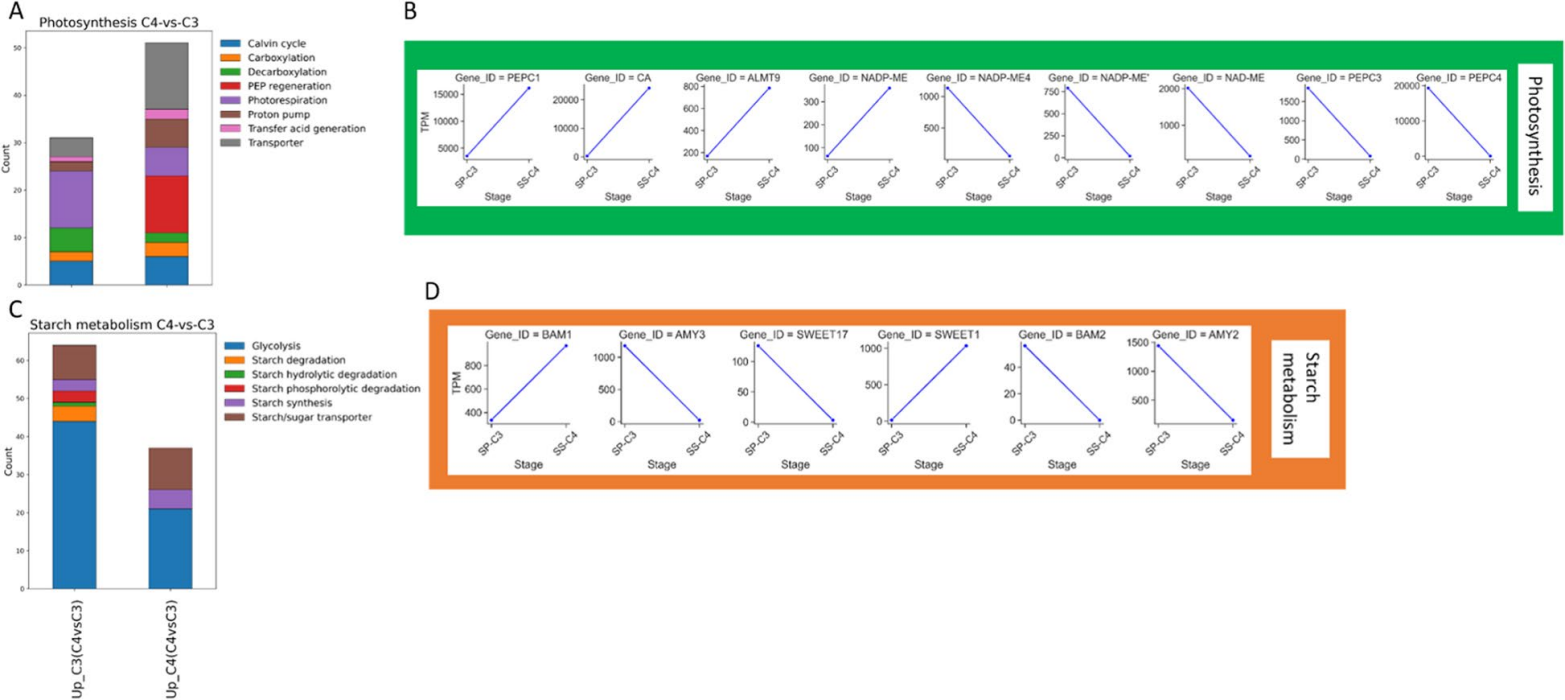
Functionally annotated DETS between C_3_ and C_4_ species. **A** Stacked bar charts showing the number of functional annotated DETS involved in CCM between C_3_ and C_4_ species. **B** Abundance of selected DETS involved in CCM between C_3_ and C_4_ species. **C** Stacked bar charts showing the number of functional annotated DETS involved in starch metabolism between C_3_ and C_4_ species. **D** Abundance of selected DETS involved in starch metabolism between C_3_ and C_4_ species. The stacked bar charts display all transcripts that were differentially expressed

Functional annotations of DETs to land plant protein sequences were assigned using Mercator4. To explore the difference between ancestral C_3_ photosynthesis to C_4_-like photosynthesis in *S. sesuvioides*, we compared the expression profile of *S. portulacastrum* (C_3_) and *S. sesuvioides* (C_4_-like) with respect to CCM. According to photosynthetic sub-pathways, DETs were clustered and the number of DETs associated with each pathway was plotted (Fig. 4). We found a significant accumulation of genes involved in C_4_-related pathways (except PEP regeneration that was only found in the C_4_-like species) in both species (Fig. 4A, Additional file 2). The number of genes involved in carboxylation, proton pump, transfer acid generation, and transporter were higher in *S. sesuvioides* (C_4_-like; Fig. 4A, Additional file 2). Conversely, genes related to decarboxylation and photorespiration were abundant in the C_3_ species as compared to C_4_-like species.

We then investigated genes related to carboxylation and decarboxylation. Surprisingly, while *PEPC1* (TRINITY_DN0_c3_g1_i12) was up-regulated in the C_4_-like species, *PEPC3* (TRINITY_DN9611_c0_g1_i3) and *PEPC4* (TRINITY_DN14827_c0_g1_i3) were significantly expressed in the C_3_ species (Fig. 4B). *PPDK* and *PPCK1* were up-regulated in the C_4_-like species (Additional file 3). The decarboxylation enzymes chloroplastic *NADP-ME4*, *NADP-ME*, and *NAD-ME* were significantly accumulated in the C_3_ species. However, another *NADP-ME* copy was up-regulated in the C_4_-like species. These findings suggest that *S. sesuvioides* as C_4_-like species employs NADP-ME as a decarboxylation enzyme but can additionally use NAD-ME (Additional file 3).

**Fig. 5:**
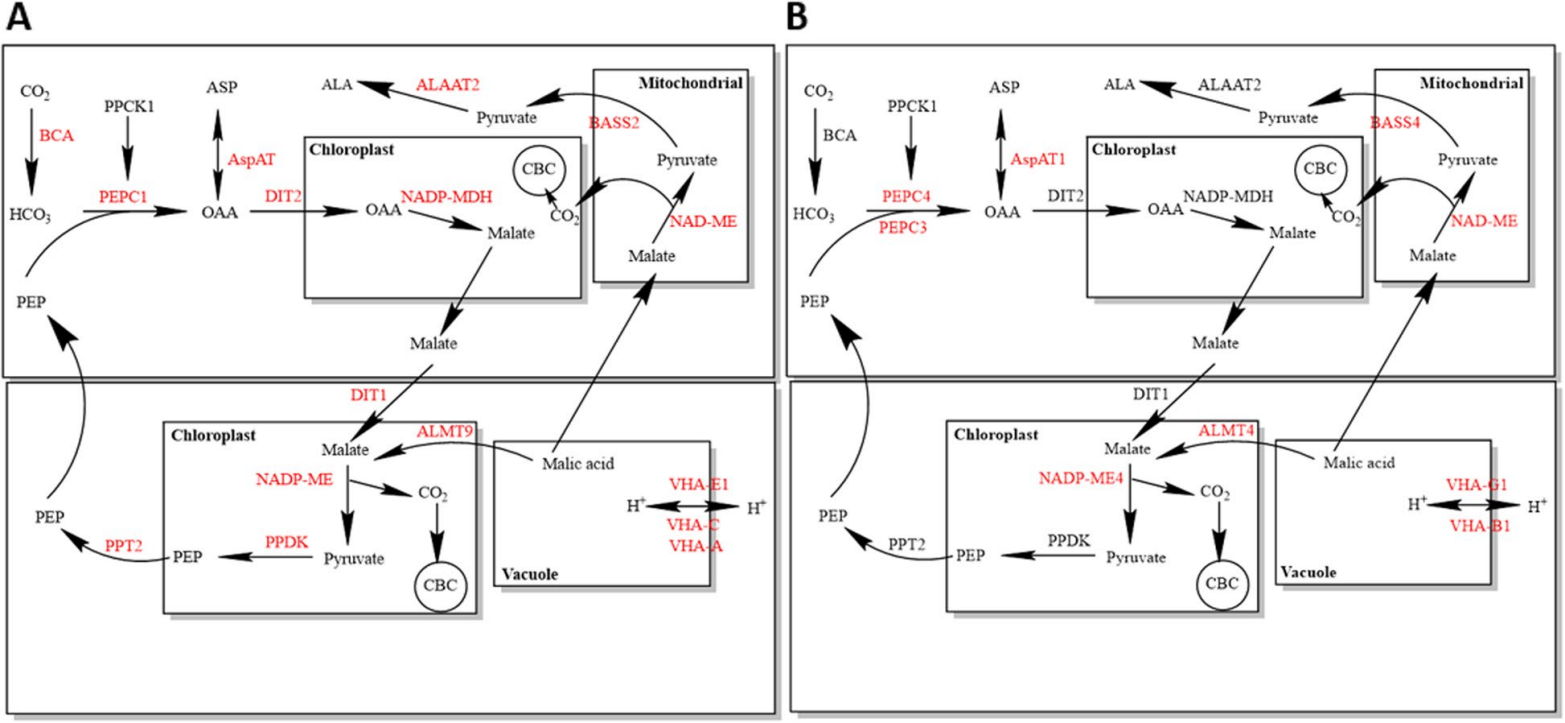
Putative photosynthetic cycle in *S. sesuvioides* **A** and *S. portulacastrum* **B**. Red colour stands for genes that were up-regulated

Gene ontology (GO) enrichment showed that response to stress was among the top 20 categories that were enriched in both species (Additional file 4). Moreover, we found that *ALMT9* and *ALMT4* were significantly up-regulated in *S. sesuvioides* and *S. portulacastrum,* respectively (Fig. 4B, Additional file 3). A putative photosynthetic cycle was designed for *S. sesuvioides* and *S. portulacastrum* (Fig. 5). Several copies of transcripts of genes that control the tonoplast potential were significantly up-regulated in *S. sesuvioides* (*VHA-A*, *VHA-C*, *VHA-E1*) and in *S. portulacastrum* (*VHA-G1*, *VHA-B1*) (Fig. 5, Additional file 3). Moreover, genes involved in starch phosphorolytic degradation were significantly abundant in *S. portulacastrum* (Fig. 4C). These genes included *AMY2* and *AMY3* (Fig. 4D).

### Functional annotation of DETs between leaves and cotyledons of *S. sesuvioides*

**Fig. 6:**
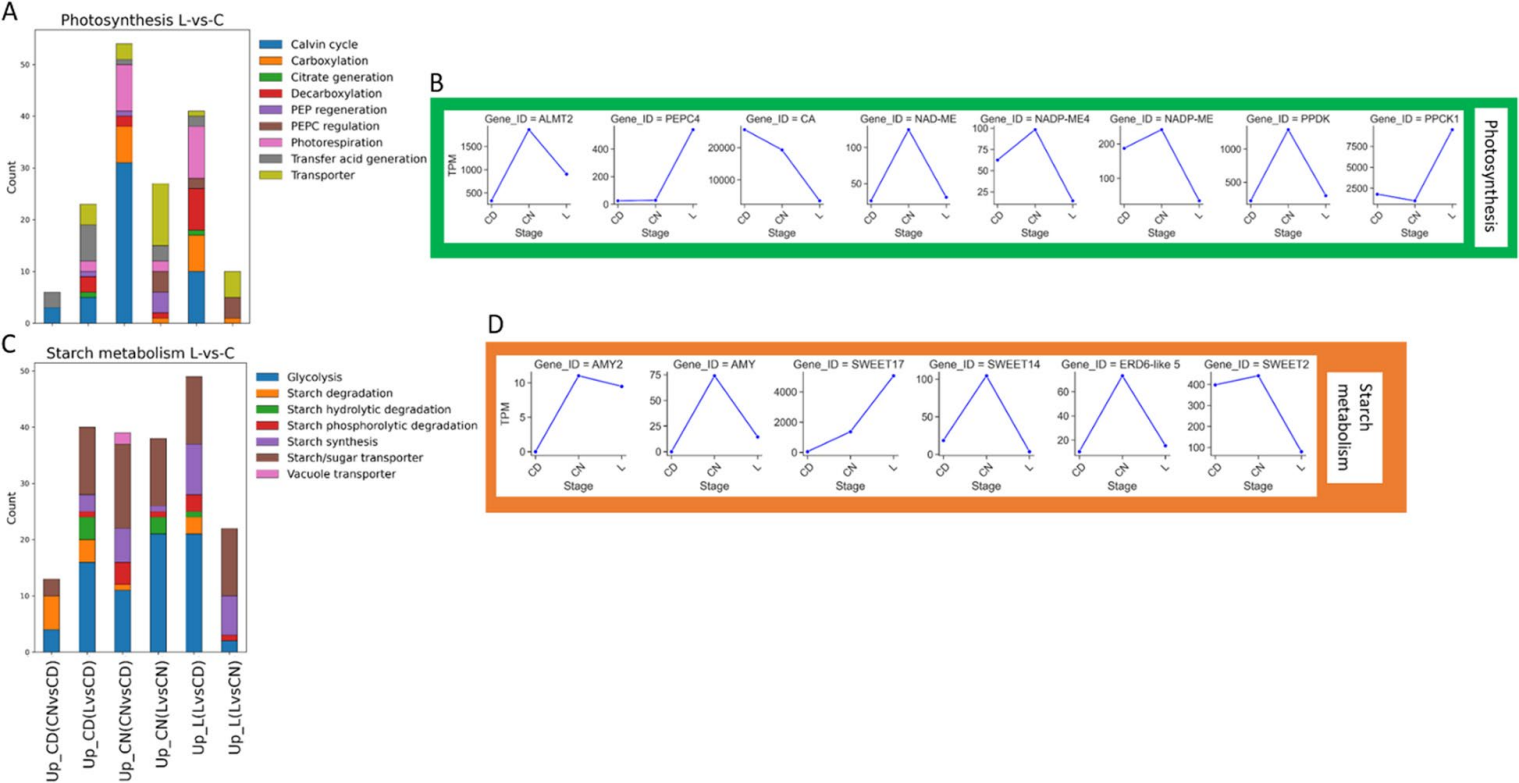
Functional annotated DETS between leaves (L) and cotelydons (C) of *S. sesuvioides*. **A** Stacked bar charts showing the number of functional annotated DETS involved in CCM between L and C of *S. sesuvioides*. **B** Abundance of selected DETS involved in CCM between L and C of *S. sesuvioides*. **C** Stacked bar charts showing the number of functional annotated DETS involved in starch metabolism L and C of *S. sesuvioides*. **D** Abundance of selected DETS involved in starch metabolism L and C of *S. sesuvioides*. The stacked bar charts display all transcripts that were differentially expressed

We examined the process of photosynthesis in the leaves and cotyledons of *S. sesuvioides*. We identified DE transcripts (*p* < 0.05, log_2_FC > 1) related to CCM according to annotation (Fig. 6, Additional file 2). The transcripts related to CCM were subsequently clustered based on their role in decarboxylation, Calvin cycle, carboxylation, citrate generation, PEP regeneration and regulation, photorespiration, transfer acid generation, and transporter. We then counted the number of related transcripts of each cluster to identify the photosynthetic mode of cotyledons and leaves (Fig. 6A, Additional file 2). We found that transcripts involved in Calvin cycle, photorespiration, carboxylation, and decarboxylation were abundant in cotyledons as compared to leaves, whereas transporters involved in CCMs (C_4_ and CAM), transfer acid generation and PEP regeneration were higher in leaves than in cotyledons (especially those collected during the day). This suggests that different CCMs are acting in leaves and cotyledons of *S. sesuvioides*. To investigate further, we plotted the abundance of transcripts associated with the functional categories. CAM differs from C_4_ by a nocturnal CO_2_ fixation and accumulation of malate or citrate in the vacuole, and a diurnal decarboxylation of accumulated malate by the malic enzymes (e.g. *NADP-ME*). Our results showed that transcripts encoding carboxylation enzymes (*PEPC4* and *PPCK1*) were upregulated in leaves as compared to cotyledon collected during the day (CD), while transcripts encoding decarboxylation enzymes *NADP-ME* and chloroplastic *NADP-ME4* were significantly abundant in cotyledons collected during the night (CN) (Fig. 6B). Moreover, phylogenetic analysis showed that *PEPC4* (TRINITY_DN14827_c0_g1_i3) grouped with a *PPC-1E1* gene, along with several *PPC-1E2* and *PPC-2* genes (Additional file 5). These results indicate a higher decarboxylation rate in CD and CN. Thus, we suspected the possibility of CAM and C_4_-like photosynthesis in cotyledons and leaves of *Sesuvium sesuvioides*, respectively.

To determine whether CAM is occurring in the cotyledons, we compared the transcript profiles of CD and CN (Fig. 6A). In CAM plants, malate generated at night is supposedly transported to the vacuole by a malate transporter aluminium-activated malate transporter (*ALMT*) [42]. We observed a significant increase in transcripts of *ALMT2* in CN (Fig. 6B). The vacuolar malate influx is driven by the difference in membrane potential established by vacuolar-type proton adenosine triphosphatase ATPase (*VHA*) and the pyrophosphate-energized membrane proton pump (*AVP*) [43]. However, as in *Portulaca* [10], no significant expression of these genes was observed in the CN. Although no significant expression of decarboxylation enzymes *NADP-ME* in CN when compared to CD, a mitochondrial *NAD-ME* was highly abundant in CN (Fig. 6B). However, we found that *RUBISCO* small unit (*RBCS1*) (Additional file 3) was up-regulated in CD. This suggested that the decarboxylation activity of *NAD-ME* at night is aimed primarily at malate respiration [44]. Additionally, ATP-citrate synthase alpha chain protein 3 (*ACL-3*) which is involved in citrate synthesis [45] was found up-regulated in CN. Cotyledons showed lower citrate levels at 11 am compared to leaves of *S. sesuvioides* (Siadjeu, unpublished data). Citrate is produced when acetyl CoA reacts with OAA suggesting that citrate contributes as well to the acidification in *S. sesuvioides* at night. Facultative CAM species such as *Talinum triangulare* exhibit increased citrate levels during the night [46].

Another feature of CAM photosynthesis is the nightly regeneration of PEP via phosphorolytic starch degradation [47, 48]. Moreover, Moreno-Villena et al. [10] hypothesized that CAM induction is associated with the increase in sugar transporter. The number of transcripts related to starch/sugar transporter and starch phosphorolytic degradation was high in CN (Fig. 6C, Additional file 2). We found that genes encoding the probable alpha-amylase 2 (*AMY2*), alpha-amylase (*AMY*) and alpha-1,4 glucan phosphorylase L-2 isozyme (*PHO2*) involved in starch phosphorolytic degradation were significantly abundant at night (Fig. 6D, Additional file 2). The sugar transporters, *SWEET14*, *SWEET17*, *ERD6-like 5* were up-regulated at night (Fig. 6D). It is worth mentioning that we did not observe a significant expression of *PEPC* at night. However, pyruvate phosphate dikinase (*PPDK*) was significantly abundant in the cotyledons at night (Fig. 3D). The enzyme PPDK catalyzes the regeneration of the CO_2_ acceptor PEP via pyruvate [49].

### Regulation and hormonal signaling in *Sesuvium*

**Fig. 7:**
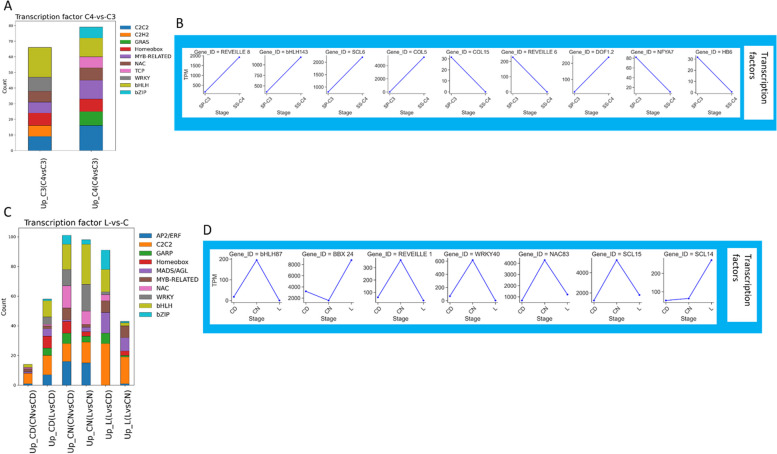
Functional annotated expressed genes related to TFs. **A** Stacked bar charts showing the number of functional annotated DET related to TFs between C_3_ and C_4_ species. **B** Abundance of selected DET related to TFs between C_3_ and C_4_ species. **C** Stacked bar charts showing the number of functional annotated DET related to TFs between (L) and cotelydons (C) of *S. sesuvioides. ***D** Abundance of selected DET related to TFs between L and C of *S. sesuvioides*. *BBX: B-BOX, bZIP: basic LEUCINE ZIPPER, CEPR: C-TERMINALLY ENCODED PEPTIDE RECEPTOR, COL: CONSTANS-LIKE, HB: HOMEOBOX, NAC (NAM, ATAF and CUC), NFYA: NUCLEAR TRANSCRIPTION FACTOR Y SUBUNIT ALPHA, SCL: SCARECROW-LIKE*. The stacked bar charts display all transcripts that were differentially expressed

To identify potential regulation and signaling elements, we performed an unsupervised k-means clustering. The three methods used showed the best k was two (Additional file 6). We found that almost all transcripts involved in photosynthesis, starch metabolism, transcription factor, and phytohormone signaling were grouped together (Cluster with the highest number of transcripts) for all comparisons (Additional files 7–8). To identify possible candidates involved in the regulation of the CCMs studies, we clustered TFs found in groups including photosynthesis, starch metabolism, and phytohormone signaling according to their families (Fig. 7, Additional file 2).

For comparison between *S. sesuvioides* and *S. portulacastrum*, TF families *C2C2*, *C2H2*, *GRAS*, *HOMEOBOX*, *MYB-related*, *NAC*, *TEOSINTE BRANCHED1/CYCLOIDEA/PROLIFERATING CELL FACTOR* (*TCP*), *WRKY*, *basic HELIX-LOOP-HELIX* (*bHLH*), and *basic LEUCINE ZIPPER* (*bZIP*) were found on top of the list (Fig. 7A, Additional file 2). Except for TFs from the GRAS family present only in C_4_-like, all others were found in C_4_-like and C_3_ species (Additional file 3). We selected based on the literature candidate TFs that are potentially involved in the regulation of C_4_ and CAM (Fig. 7B). Different isoforms are recruited in the regulation of C_3_ and C_4_-like species. Our data showed that *REVEILLE 8*, *bHLH143*, *SCL6*, *COL5*, and *DOF1.2* were significantly up-regulated in C_4_-like species, whereas *REVEILLE 6*, *COL15*, *NFYA7*, and *HB6* were up-regulated in the C_3_ species (Fig. 7B, Additional file 3). We identified 63 nonredundant motifs (*p* < 0.01, E-value < 0.05) in sequences of genes upregulated in the C_4_-like species while 36 motifs were found in the C_3_ species (Additional file 9). The top five of the most enriched motifs were annotated to *C2C2-DOF* families in C_4_-like and the C_3_ species with the element CTTTTT (Table 2). Although the most enriched motifs were similar between the C_4_-like species and the C_3_ species, the second most enriched motifs were elements (*GAGA*, *BBR/BPC* family) and (CACCAACM, *MYB* family) in the C_4_-like and C_3_ species, respectively. We found several motifs often present in the same transcript sequences. This suggests a coordinated and regulatory network of TFs controlling photosynthesis in which motif CTTTTT is dominant.

**Table 2:** Top five motif enrichments of differentially expressed genes related to CCM (Carbon Concentrating Mechanism) and starch metabolism in *Sesuvium* species

	**Rank**	**JASPAR matrix ID**	**Associated TF**	**Consensus sequence**	**E-value**	**Occurrence**
Up_L(LvsCD)	1	MA1274.1	*DOF3.6*	TTTWCTTTTTHHYTTTTTTTT	2.06E-25	39
	2	MA1267.1	*DOF5.8*	WHTTTTTTHYTTTTTACTTTTTNHTTTWW	1.88E-21	45
	3	MA1268.1	*CDF5*	TTTTYACTTTTTYTTTTTTTTTTTTTW	2.65E-19	34
	4	MA1281.1	*DOF5.1*	RAAAAAGWAAAAARAAAAARA	3.11E-16	36
	5	MA1277.1	*DOF1.7*	AAAAAVAAAAAGTARAAAAWR	2.71E-13	43
Up_CD(LvsCD)	1	MA1274.1	*DOF3.6*	TTTWCTTTTTHHYTTTTTTTT	1.96E-21	34
	2	MA1267.1	*DOF5.8*	WHTTTTTTHYTTTTTACTTTTTNHTTTWW	1.06E-19	45
	3	MA1823.1	*Zm00001d027846*	RRAAAGAAAARR	8.96E-15	51
	4	MA1281.1	*DOF5.1*	RAAAAAGWAAAAARAAAAARA	1.14E-13	41
	5	MA1404.1	*BPC1*	GAGAGAGAGAGAGAGAGAGAGAGA	1.61E-13	21
Up_CN(CDvsCN)	1	MA1274.1	*DOF3.6*	TTTWCTTTTTHHYTTTTTTTT	3.07E-19	26
	2	MA1268.1	*CDF5*	TTTTYACTTTTTYTTTTTTTTTTTTTW	1.01E-13	26
	3	MA1267.1	*DOF5.8*	WHTTTTTTHYTTTTTACTTTTTNHTTTWW	1.03E-11	20
	4	MA1815.1	GRF4*GRF4*	HDGCAGCAGCWDY	3.78E-11	41
	5	MA1281.1	DOF5.1*DOF5.1*	RAAAAAGWAAAAARAAAAARA	1.07E-10	21
Up_CD(CDvsCN)	1	MA1267.1	*DOF5.8*	WHTTTTTTHYTTTTTACTTTTTNHTTTWW	2.04E-06	14
	2	MA1268.1	*CDF5*	TTTTYACTTTTTYTTTTTTTTTTTTTW	1.31E-04	10
	3	MA1277.1	*DOF1.7*	AAAAAVAAAAAGTARAAAAWR	3.30E-04	13
	4	MA1274.1	*DOF3.6*	TTTWCTTTTTHHYTTTTTTTT	6.38E-04	7
	5	MA1262.1	*ERF2*	YCDCCDCCDCCGCCGCCRYYD	7.36E-04	8
Up_C_3_(C_4_vsC_3_)	1	MA1404.1	*BPC1*	GAGAGAGAGAGAGAGAGAGAGAGA	3.45E-19	23
	2	MA1274.1	*DOF3.6*	TTTWCTTTTTHHYTTTTTTTT	1.15E-17	40
	3	MA1402.1	*BPC6*	CTCTCTCTCTCTCTCTCTCTC	1.24E-16	18
	4	MA1281.1	*DOF5.1*	RAAAAAGWAAAAARAAAAARA	1.52E-12	42
	5	MA1267.1	*DOF5.8*	WHTTTTTTHYTTTTTACTTTTTNHTTTWW	7.37E-11	48
Up_C_4_(C_4_vsC_3_)	1	MA1274.1	*DOF3.6*	TTTWCTTTTTHHYTTTTTTTT	1.64E-26	31
	2	MA1281.1	*DOF5.1*	RAAAAAGWAAAAARAAAAARA	7.24E-19	23
	3	MA1267.1	*DOF5.8*	WHTTTTTTHYTTTTTACTTTTTNHTTTWW	4.42E-16	20
	4	MA1404.1	*BPC1*	GAGAGAGAGAGAGAGAGAGAGAGA	9.93E-16	20
	5	MA1268.1	*CDF5*	TTTTYACTTTTTYTTTTTTTTTTTTTW	3.88E15	24

Our results indicated that the same TF families are recruited to a variable degree for controlling cotyledons and leaves of *S. sesuvioides*. The top 10 TF families abundant in cotyledons and leaves during the day and in CN were *bHLH*), C2C2, A*PETALA2*/*ETHYLENE-RESPONSIVE FACTOR* (*AP2/ERF*), *WRKY*, *MADS/AGL*, *bZIP*, *NAC*, *MYOLOBLASTOSIS* (*MYB*)*-related*, *GARP* (*GOLDEN2*, *ARR-B, PSR1*), and *HOMEOBOX* (Fig. 7C, Additional file 2). While during the day, the number of TFs related to *C2C2*, *bHLH*, and *bZIP* families were higher in leaves than in cotyledons, no TFs related to *HOMEOBOX* and *AP2/ERF* were enriched in leaves. When comparing CD and CN, we found nearly all TF families were abundant at night. We then specifically looked at TFs that are frequently expressed in these families. Genes were selected by their potential involvement in the regulation of CAM and C_4_-like photosynthesis. Our data showed the TFs *bHLH87*, *REVEILLE1*, *WRKY40*, *NAC83*, and *SCL15* were up-regulated in the CN, whereas TFs *BBX24*, and *SCL14* were significantly abundant in the leaves (Fig. 7D). To confirm whether these TFs regulate C_4_-like and CAM photosynthesis, we explored TF binding sites in sequences of transcripts related to C_4_-like photosynthesis and starch metabolism (Table 2). Motif enrichment analysis revealed elements AAAAAG and CTTTTT from the *C2C2-DOF* family were the most enriched in leaves during the day, while element CTTTTT was the most enriched in cotyledons (Table 2). When comparing CD and CN, element CGCCGCC from the *AP2/ERF* family was enriched in CD whereas element (CTTTTT) was enriched in all comparisons.

**Fig. 8:**
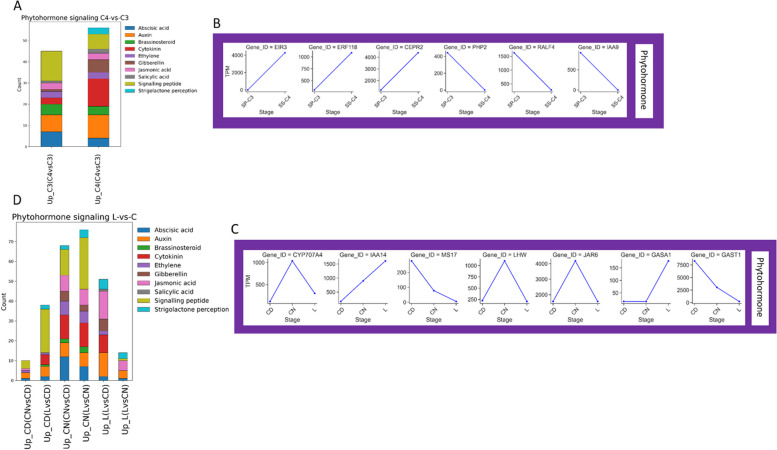
Functional annotated differentially expressed genes related to phytohormones. **A** Stacked bar charts showing the number of functional annotated DET related to phytohormones between C_3_ and C_4_ species. **B** Abundance of selected DET related to phytohormones between C_3_ and C_4_ species. **C** Stacked bar charts showing the number of functional annotated DET related to phytohormones between (L) and cotelydons (C) of *S. sesuvioides*. **D** Abundance of selected DET related to phytohormones between (L) and cotelydons (C) of *S. sesuvioides*. *CEPR: C-TERMINALLY ENCODED PEPTIDE RECEPTOR, CYP707A4: CYTOCHROME P450, EIR: ETHYLENE INSENSITIVE ROOT, ERF: ETHYLENE-RESPONSIVE ELEMENT BINDING*

Phytohormones play critical roles in photosynthesis regulation and developmental processes ranging from organ initiation to senescence [50]. Moreover, phytohormones have been shown to mediate TF action in C_4_ and CAM plants [51]. When comparing *S. sesuvioides* to *S. portulacastrum*, we found that signaling peptides, auxin, cytokinin, abscisic acid, and brassinosteroid were the top five endogenous signaling hormones (Fig. 8A, Additional file 2). There were many genes related to hormonal signals that were specific to either C_3_ species or C_4_-like species (Additional file 3). For instance, genes *PHP2* (cytokinin), *RALF4* (signaling peptide), and IAA9 (auxin) were significantly accumulated in the C_3_ species while *EIR3* (auxin), *ERF118* (cytokinin) and *CEPR2* (signaling peptide) were significantly accumulated in the C_4_-like species (Fig. 8B). When comparing leaves to cotyledons, signaling peptides, auxin, cytokinin, jasmonic, and abscisic acids were the most abundant signaling hormones (Fig. 8C, Additional file 2). We found that the expression of *CYP707A4* (abscisic acid), *LHW* (cytokinin), and *JAR6* (jasmonic acid) increased significantly in abundance in cotyledons at night (Fig. 8D). In CD, *MS17* (Auxin) and *GAST1* (signaling peptide) were up-regulated, while *IAA14* (auxin) and *GASA1* (signaling peptide) were significantly accumulated in the leaves. Many other signaling protein genes were found specific to leaves and cotyledons and are listed in Additional file 3.

## Discussion

### Photosynthetic mode in *Sesuvium* species

Our findings confirmed that adult leaves of *S. sesuvioides* perform C_4_-like photosynthesis with core C_4_ enzymes up-regulated. These up-regulated enzymes were involved in carboxylation (*βCA*, *PEPC1*), acid regeneration (*ALAAT2*, *ASPAT*, *NADP-MDH*), decarboyxlation (*NADP-ME*, *NAD-ME*), transporters (*BASS2*, *DIT1*, *DIT2*, *PPT2*) and PEP regeneration (*PPDK*). *PEPC1* (TRINITY_DN0_c3_g1_i12) was clustered in the group containing only *PPC-1E1* (Additional file 5) which was repeatedly used for both C_4_ and CAM photosynthesis [20]. This result is in accordance with anatomical, biochemical, and physiological observations [17]. The C_4_-like status of *S. sesuvioides* becomes evident in the still relatively high expression of photorespiratory genes (Fig. 3C, Additional file 2).

Intriguingly, when comparing leaves of the adult plants with cotyledons of *S. sesuvioides*, the main carboxylation enzyme was PEPC isoform 4 (*PEPC4*) in leaves. There are several plant *PEPC* copies that are classified into photosynthetic (C_4_ and CAM) and non-photosynthetic isoforms [52]. In eudicots, there are three distinct lineages that encode *PEPC*, which are called *PPC-1E1*, *PPC-1E2*, and *PPC-2* [20]. According to the Uniprot database, these genes are involved in CO_2_ fixation and the tricarboxylic acid cycle. Phylogenetic analysis of PEPC4 with these three genes from 35 species (Additional file 5) showed that *PEPC4* (TRINITY_DN14827_c0_g1_i3) clustered together with a *PPC-1E1*, as well as several *PPC-1E2* and *PPC-2* genes. However, the presence of *PPC-1E1* in this group suggests that PPC4 may be involved in C_4_ photosynthesis. Indeed, *PEPC4* isoform identified and up-regulated in the leaves is homologous to Arabidopsis *AtPEPC4* which is involved in photosynthesis (https://www.uniprot.org/uniprotkb/Q8GVE8/entry) and may indicate a similar role in *S. sesuvioides*. In line with this result, *PEPC3* and *PEPC4* were significantly expressed in adult C_3_ leaves of *S. portulacastrum* when compared to the leaves of C_4_-like *S. sesuvioides*. *PEPC3* (TRINITY_DN9611_c0_g1_i3) was also found clustered in a similar group with *PEPC4*. This could also be explained by the fact that the C_3_ species *S. portulacastrum* induces weak CAM photosynthesis under drought stress [15]. Heat and drought stresses repress nitrogen metabolism enzymes [53]. Thus, regulation of nitrogen metabolism is crucial to maintain plant growth under stress conditions. *PEPC* plays a crucial role in C_4_ photosynthesis and in modulating the balance of carbon and nitrogen metabolism in *Arabidopsis* [54]. This indicates that the up-regulation of these *PEPC* copies may be important to maintain the photosynthetic system and plant growth of *Sesuvium* species under adverse conditions. The biological role and localization of these *PEPC* copies in *Sesuvium* need to be further investigated, however, our results indicate that different *PEPC* copies are optimized for different photosynthetic functions in leaves and cotyledons of *Sesuvium*.

Generally, there are three subtypes of C_4_ photosynthesis depending on the decarboxylation enzymes. The main decarboxylation enzyme in *S. sesuvioides* was *NADP-ME*. The up-regulation of *NADP-ME* indicates that *S. sesuvioides* is employing *NADP-ME* in its decarboxylation mechanism. This result is consistent with biochemical and physiological observations in *S. sesuvioides* [17]. Moreover, in the C_3_ species, multiple copies of the chloroplastic decarboxylation enzymes *NADP-ME* and *NAD-ME* were significantly accumulated. These findings may indicate a possibility of CAM induction in *S. portulacastrum*.

It was shown indeed that *S. portulacastrum* is capable of inducing CAM in stressful conditions [15,55]. *ALMT9* and *ALMT4* were significantly up-regulated in *S. sesuvioides* and *S. portulacastrum,* respectively. *ALMT* was originally responsible for nocturnal malate accumulation caused by an inward-rectifying anion-selective channel that forces only malate influx to the vacuole [56]. However, Meyer et al. [57] demonstrated that *AtALMT6* functions as a malate influx or efflux channel depending on the tonoplast potential. This indicates diurnal vacuolar malate efflux in both species, hence the possibility of weak CAM being induced in *S. sesuvioides* and *S. portulacastrum*.

### Photosynthetic plasticity in *Sesuvium*

Plants exhibit plasticity for a wide variety of ecologically important traits to adjust to environmental changes [58]. Photosynthetic plasticity underpins the ability of plants to acclimate and grow in adverse environments and may depend on plant ontogeny. Our data provide evidence of photosynthetic plasticity in *S. sesuvioides* with C_4_-like and CAM photosynthesis in leaves and cotyledons, respectively. During the day, decarboxylating enzymes were more strongly expressed in cotyledons compared to leaves while carboxylating enzymes were strongly expressed in leaves. This result was further confirmed by titratable acidity, which showed a significant accumulation of acids overnight in cotyledons. The co-occurrence of C_4_ and CAM photosynthesis has already been reported in other Aizoaceae, namely for *Trianthema portulacastrum* [9], but this is the first time to report ontogenetic variability with respect to photosynthesis in the Aizoaceae family with CAM in cotyledons and C_4_-like in leaves. In Amaranthaceae (incl. Chenopodiaceae), Lauterbach et al. [59] based on RNA expression profiles showed the transition from C_3_ photosynthesis in cotyledons to C_4_ photosynthesis in adult leaves of *Salsola soda*. This phenomenon seems to occur in several species of Salsoleae according to C_3_-like features such as lower carbon isotope ratios and lack of Kranz anatomy in cotyledons (e.g., [60, 61]). However, these species have never been tested for CAM metabolism. The presence of CAM in cotyledons may be induced by environmental cues. Indeed, no CAM was observed in the cotyledons and leaves of *S. sesuvioides* under well-watered conditions [17]. In the climate chamber, stressful conditions were mainly created by the maximum light intensity. CAM induction has been linked to a photoprotective role in *Portulaca oleracea* [51]. This suggests a photoprotective role of CAM induction in cotyledons of *S. sesuvioides*. It is worth mentioning that a significant expression of *PEPC* (Phosphoenolpyruvate carboxylase) was not observed in CN. This is likely due to the sampling time (one hour after the light was turned off). However, a significant abundance of *PPDK* was observed in CN. This enzyme catalyzes the regeneration of PEP via pyruvate, which serves as a CO_2_ acceptor.

### Integration of C_4_-like and CAM photosynthesis

Our data suggested a possible co-occurrence of C_4_-like and CAM photosynthesis in a single leaf of *S. sesuvioides* under adverse conditions (Fig. 5, Fig. 6A). In CAM photosynthesis, nocturnally accumulated malate is translocated out of the vacuole by a malate channel for subsequent decarboxylation during the light period. In *P. oleracea*, a C_4_ species that performs CAM when drought-stressed [10], *AtALMT9* that has been associated with CAM function [62] is a vacuolar malate channel [63]. Interestingly, we found *ALMT9* was significantly abundant during the light period in *S. sesuvioides*. Thus, the up-regulation of *ALMT9* in leaves of *S. sesuvioides*, suggests that *ALMT9* may function as a vacuolar malate efflux channel in *S. sesuvioides* and is therefore linked to CAM function.

Taking all results together, this may imply the integration of the hybrid system C_4_-like + CAM in *S. sesuvioides* under stress conditions. However, the modularity of this integration needs to be investigated. This co-occurrence of C_4_-like and CAM in a single leaf in *S. sesuvioides* is probably facilitated by the particular C_4_-like phenotype of *S. sesuvioides* leaves where Rubisco is present in the MCs. The MCs of *S. sesuvioides* are succulent and outnumber the Kranz cells by two-fold. When the leaves grow older, the M portion becomes even larger and the carbon isotope ratios drop [17]. This might indicate a photosynthetic plasticity towards a higher proportion of CAM and or C_3_ relative to C_4_-like in older leaves depending on the growing conditions.

### Regulation of photosynthesis and hormonal signaling in *Sesuvium*

Plants have the ability to choose different photosynthetic pathways, which is controlled by TFs. Six TF families i.e., *C2C2*, *HOMEOBOX*, *NAC*, *WRKY*, *bHLH*, and *bZIP* were found up-regulated in leaves and cotyledons of *S. sesuvioides* when compared leaves to cotyledons and also when compared to adult leaves of *S*. *sesuvioides* and to adult leaves of *S. portulacastrum*. These families have been hypothesized to be involved in the regulation of C_4_ and CAM photosynthesis in Chenopodiaceae, Aizoaceae, and Asteraceae [51,64]. However, TFs from the *C2C2*, and *bHLH* families were the most expressed in leaves, and cotyledons during day and night. Likewise, these TFs were predominant in the C_3_ and C_4_-like species. This may indicate the significant weight of the *C2C2* and *bHLH* TF families in the regulation of cotyledons and leaf development in *S. sesuvioides* and *S. portulacastrum*. The binding site of TF C_4_*ZINC FINGER-TYPE* (*DOF3.6*) from the *DOF/C2H2* was most enriched in C_4_ and CAM genes in all comparisons. In addition, at least three copies of *DOFs* were among the top five. Several copies of *DOF* proteins (*DOF1* and *DOF2*) were found involved in the regulation of the light-dependent C_4_ gene PEPC in maize with antagonist effects. While *DOF1* activates C_4_ genes, *DOF2* can activate or repress them [65]. These results indicate that different copies of *DOF* genes are likely involved in the regulation of C_4_-like and CAM genes in *S. sesuvioides* and *S. portulacastrum*, as well. It is worth mentioning that transcription factors (TFs) from the *MYB*, *NUCLEAR FACTOR Y* (*NF-Y*) and NAC families have been suggested to play a role in regulating CAM in facultative CAM species such as *Mesembryanthemum crystallinum* [66] and *T. triangulare* [67]. While the isoforms of these genes may differ, these TF families were found to be up-regulated when comparing CAM-induced (CD) and non-induced (CN) conditions, as well as when comparing day-time leaves and cotyledons.

Transcription factors are regulated by phytohormones (signaling molecules) under environmental stresses [68]. Our data showed that phytohormones were clustered with C_4_ and CAM genes, as well as TFs which indicates a regulatory network involving TFs and phytohormones. Indeed, Ferrari et al. [51] found that *ABA* and *CK-related* genes regulate TFs connected to CAM and C_4_ photosynthesis in *Portulaca oleracea*. It has been suggested that *ABA* plays a role in responding to drought stress in facultative CAM species such as *M. crystallinum* [69] and *T. triangulare* [46,67]. In *S. sesuvioides* cotyledons, transcripts of genes that encode for *ABA* were found to be more enriched in CD as compared to CN. As the temperature at night was cooler than during the day, this could indicate that *ABA* might also play a role in CAM induction. While ABA and CKs have been studied intensely in CAM and C_4_ photosynthesis, several other phytohormones that regulate photosynthesis (reviewed by [50]) have received little attention. Here, we found that transcripts encoding signaling peptides were the most abundant plant hormones during the day and at night in cotyledons as opposed to leaves. Similarly, diurnal and nocturnal comparison expression in cotyledons showed that signaling peptides were predominantly accumulated. Transcripts of genes encoding for the signaling peptides Gibberellic acid *(GA)-STIMULATED ARABIDOPSIS/GA-STIMULATED TRANSCRIPT* (*GAST*) and the *RAPID ALKALINIZATION FACTOR* (*RAFL*) were predominantly expressed in cotyledons as compared to leaves. These plant hormones play important roles in plant growth, development, and stress responses ( [70, 71]), and may control cotyledon growth and response to environmental conditions in *S. sesuvioides*. Conversely, in leaves as opposed to CD, the most dominant hormone was jasmonic acid followed by auxin and cytokinin CKs. Jasmonic acid, auxin, and cytokinin are classical phytohormones that regulate various aspects of plant growth and abiotic and biotic stress responses. While jasmonic acid can regulate stomatal closure and opening under drought stress in *Arabidopsis* [72], auxin coordinates cell division, expansion, and differentiation [73], and CKs are implicated in cell cycle progression [74]. Several genes encoding for these phytohormones were found to be significantly accumulated (Additional file 3) and should be used as candidate genes involved in the regulation of CCMs in these species.

## Conclusions

This study of gene expression profiles of *S. sesuvioides* provides evidence of extraordinary photosynthetic plasticity under adverse conditions with induced CAM in cotyledons and an integration of CAM and C_4_-like photosynthesis in adult leaves. However, the modularity of the co-occurrence of the two CCMs needs to be explored in future studies. We assume that further detection of co-occurring CCMs is just a matter of more experimental studies that explicitly look for this and we believe that it is more common in succulent C_4_ lineages than currently known. Our findings suggest a complex regulatory network involving TFs and phytohormones and underpin the regulation of CCMs and adaptation of *Sesuvium* species which grow in disturbed and highly dynamic environments.

### Supplementary Information

Additional file 1: Figure S1. Images of potted *S. sesuvioides* plants growing in the climate chamber and in the uncontrolled greenhouse environment.

Additional file 2: Dataset S1. List of transcripts/genes belonging to each functional group in all comparisons.

Additional file 3: Dataset S2. DE transcripts in all comparisons.

Additional file 4: Figure S2. Gene ontology enrichment.

Additional file 5: Fig. S3. Unrooted maximum likelihood tree of genes (*PPC-1E1, PPC-1E2* and *PPC-2*) from 35 different species with 11 transcripts of genes encoding *PEPC*. The IDs of *PPC-1E1*, PPC-1E2, and *PPC-2* in the tree are Uniprot IDs, these IDs can be used to retrieve the corresponding protein sequences.

Additional file 6: Fig. S4. The best K values were determined using the Elbow and Silhouette methods, as well as the Gap Statistic, in all comparisons.

Additional file 7: Fig. S5. K-means clustering of DE transcripts between leaves and cotyledons of *S. seuvioides* and between C_3_ and C_4_ species.

Additional file 8: Dataset S3. List of DE transcripts within clusters in all comparisons.

Additional file 9: Dataset S4. Motif enrichments of DE genes related to CCM (Carbon Concentrating Mechanism) and starch metabolism in *Sesuvium* species.

## Data Availability

The datasets used and/or analysed during the current study are available in the NCBI Sequence Read Archive (SRA) under the BioProject ID: PRJNA1067387, http://www.ncbi.nlm.nih.gov/bioproject/1067387
