# Supplementary material for: C4-like Sesuvium sesuvioides (Aizoaceae) exhibits CAM in cotyledons and putative C4-like + CAM metabolism in adult leaves as revealed by transcriptome analysis
Source: BMC Genomics. 2024 Jul 13;25:688. doi: 10.1186/s12864-024-10553-2 (PMC11245778; doi:10.1186/s12864-024-10553-2)
Supplement: Supplementary file 1 — Additional file 1: Figure S1. Images of potted S. sesuvioides plants growing in the climate chamber and in the uncontrolled greenhouse environment. [file 12864_2024_10553_MOESM1_ESM.pdf]

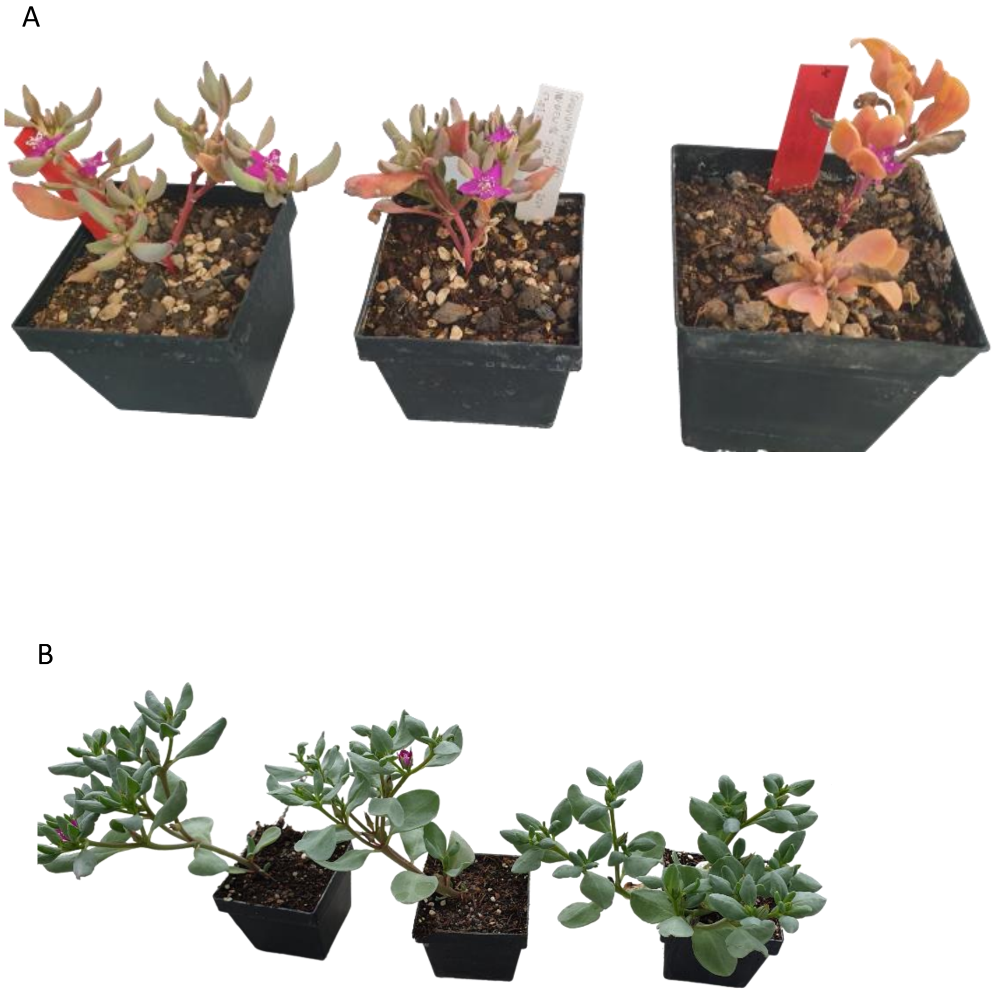

Additional file 1: Figure S1. Images of potted *S. sesuvioides* plants growing in the climate chamber and in the uncontrolled greenhouse environment. A: Stressed plants from the climate chamber, B: non stressed plants from the greenhouse.
