# Supplementary figures and images for: C4-like Sesuvium sesuvioides (Aizoaceae) exhibits CAM in cotyledons and putative C4-like + CAM metabolism in adult leaves as revealed by transcriptome analysis
Source: BMC Genomics. 2024 Jul 13;25:688. doi: 10.1186/s12864-024-10553-2 (PMC11245778; doi:10.1186/s12864-024-10553-2)

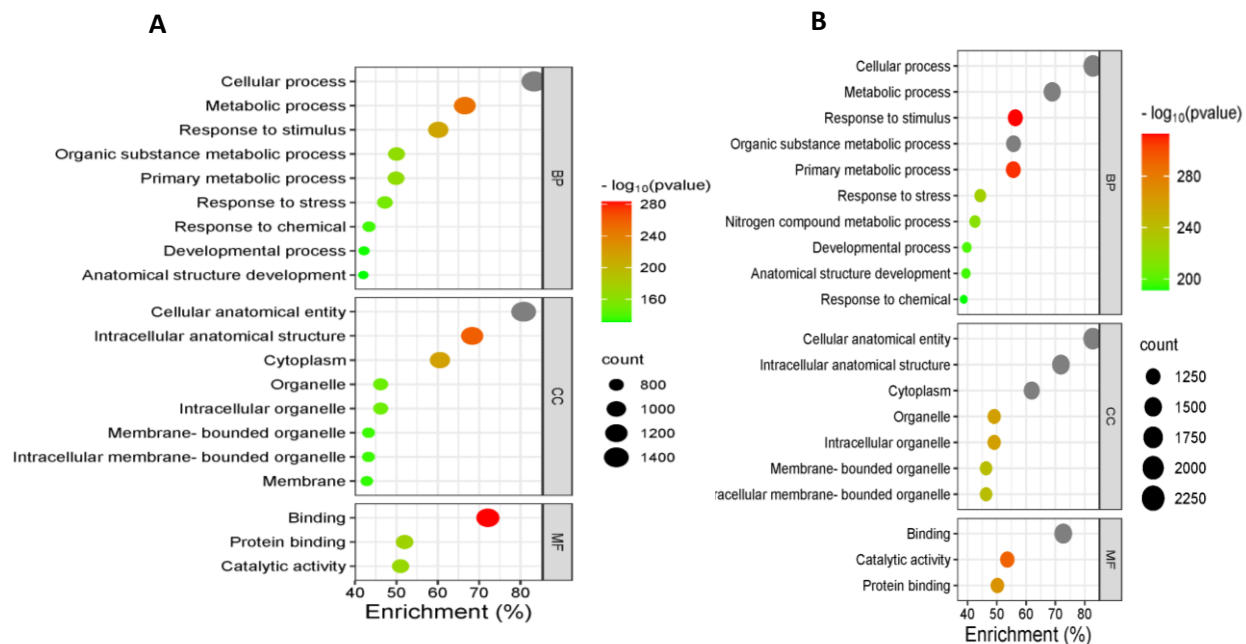

**Additional file 4: Figure S2.** Gene ontology enrichment. **(A)** *S. sesuvioides*, **(B)** *S. portulacastrum*

Supplement: Supplementary file 4 — Additional file 4: Figure S2. Gene ontology enrichment. [file 12864_2024_10553_MOESM4_ESM.pdf]
