# Supplementary material for: C4-like Sesuvium sesuvioides (Aizoaceae) exhibits CAM in cotyledons and putative C4-like + CAM metabolism in adult leaves as revealed by transcriptome analysis
Source: BMC Genomics. 2024 Jul 13;25:688. doi: 10.1186/s12864-024-10553-2 (PMC11245778; doi:10.1186/s12864-024-10553-2)
Supplement: Supplementary file 5 — Additional file 5: Fig. S3. Unrooted maximum likelihood tree of genes (PPC-1E1, PPC-1E2 and PPC-2) from 35 different species with 11 transcripts of genes encoding PEPC. The IDs of PPC-1E1, PPC-1E2, and PPC-2 in the tree are Uniprot IDs, these IDs can be used to retrieve the corresponding protein sequences. [file 12864_2024_10553_MOESM5_ESM.pdf]

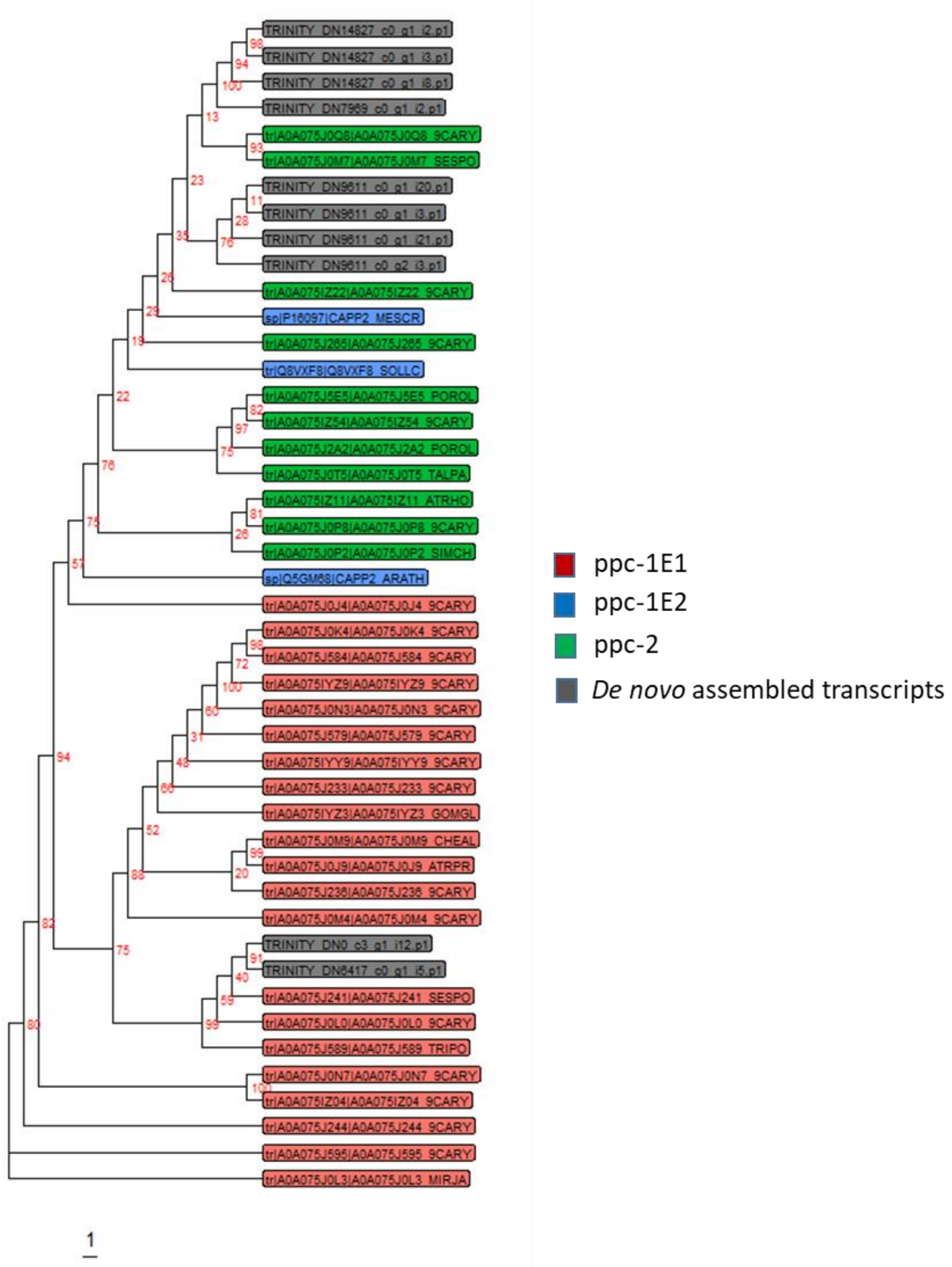

Additional file 5: Fig. S3. Unrooted maximum likelihood tree of genes (*PPC-1E1*, *PPC-1E2* and *PPC-2*) from 35 different species with 11 transcripts of genes encoding *PEPC*. The IDs of *PPC-1E1*, PPC-1E2, and *PPC-2* in the tree are Uniprot IDs, these IDs can be used to retrieve the corresponding protein sequences.
