# Supplementary material for: C4-like Sesuvium sesuvioides (Aizoaceae) exhibits CAM in cotyledons and putative C4-like + CAM metabolism in adult leaves as revealed by transcriptome analysis
Source: BMC Genomics. 2024 Jul 13;25:688. doi: 10.1186/s12864-024-10553-2 (PMC11245778; doi:10.1186/s12864-024-10553-2)
Supplement: Supplementary file 6 — Additional file 6: Fig. S4. The best K values were determined using the Elbow and Silhouette methods, as well as the Gap Statistic, in all comparisons. [file 12864_2024_10553_MOESM6_ESM.pdf]

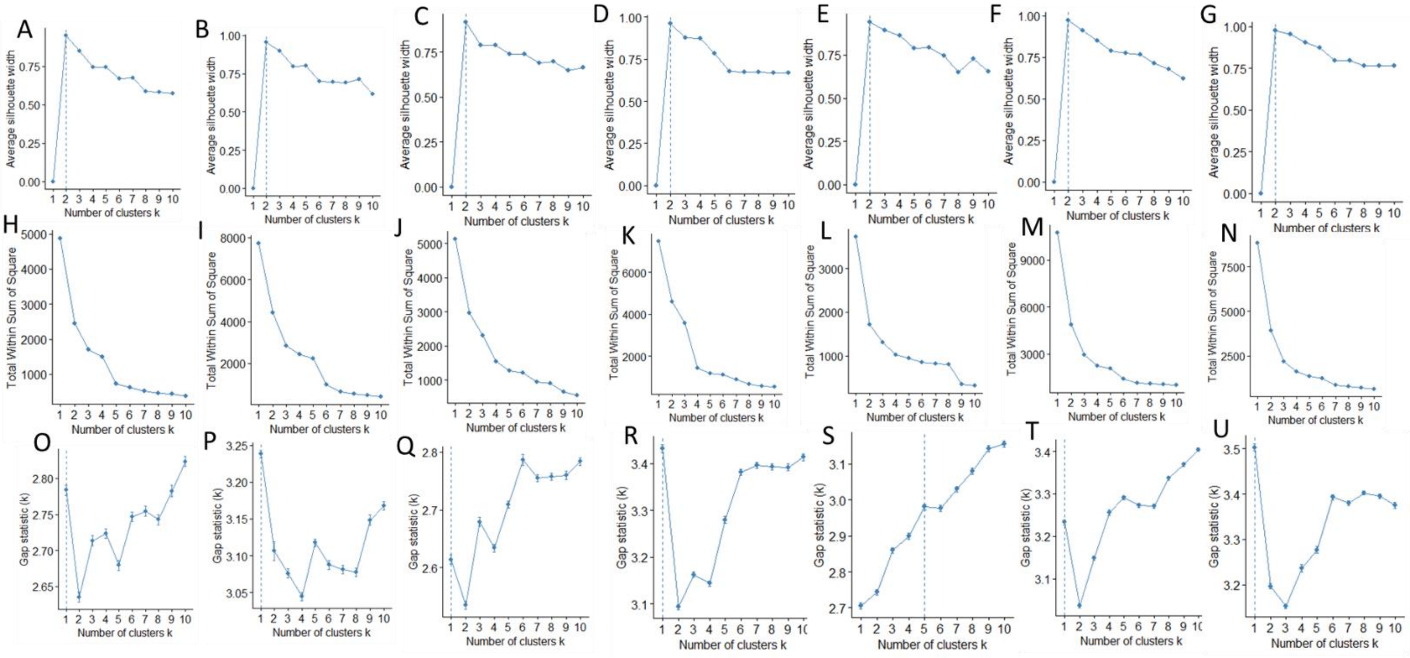

Additional file 6: Figure S4. The best K value. **(A)** Transcripts up-regulated in the C_4-like species, as compared to the C_3 species (Up_C_4/C_3_vs_C_4, the Silhouette method) **(B)** Transcripts up-regulated in the C_3 species, as compared to the C_4 species (Up_C_3/C_3_vs_C_4, the Silhouette method). **(C)** Transcripts up-regulated in cotyledons night, as compared to cotyledons day (Up_CN/CD_vs_CN, the Silhouette method) of *S. sesuvioides*. **(D)** Transcripts up-regulated in leaves, as compared to cotyledons day (Up_L/L_vs_CD, The Silhouette method) of *S. sesuvioides*. **(E)** Transcripts up-regulated in leaves, as compared to cotyledons night (Up_L/L_vs_CN, the Silhouette method) of *S. sesuvioides*. **(F)** Transcripts up-regulated in cotyledons, as compared to leaves of *S. sesuvioides* (Up_CD/L_vs_CD, the Silhouette method) **(G)** Transcripts up-regulated in cotyledons night, as compared to leaves (Up_CN/L_vs_CN, the Silhouette method) of *S. sesuvioides*. **(H)** The best K value. **(A)** Transcripts up-regulated in the C_4-like species, as compared to the C_3 species (the Elbow method) **(I)** Transcripts up-regulated in the C_3 species, as compared to the C_4 species (Up_C_3/C_3_vs_C_4, the Elbow method). **(J)** Transcripts up-regulated in cotyledons night, as compared to cotyledons day (Up_CN/CD_vs_CN, the Elbow method) of *S. sesuvioides*. **(K)** Transcripts up-regulated in leaves, as compared to cotyledons day (Up_L/L_vs_CD, The Elbow method) of *S. sesuvioides*. **(L)** Transcripts up-regulated in leaves, as compared to cotyledons night (Up_L/L_vs_CN, the Elbow method) of *S. sesuvioides*. **(M)** Transcripts up-regulated in cotyledons, as compared to leaves of *S. sesuvioides* (Up_CD/L_vs_CD, the Elbow method). **(N)** Transcripts up-regulated in cotyledons night, as compared to leaves (Up_CN/L_vs_CN, the Elbow method) of *S. sesuvioides*. **(O)** Transcripts up-regulated in the C_4-like species, as compared to the C_3 species (the Gap statistic) **(P)** Transcripts up-regulated in the C_3 species, as compared to the C_4 species (Up_C_3/C_3_vs_C_4, the Gap statistic). **(Q)** Transcripts up-regulated in cotyledons night, as compared to cotyledons day (Up_CN/CD_vs_CN, the Gap statistic) of *S. sesuvioides*. **(R)** Transcripts up-regulated in leaves, as compared to cotyledons day (Up_L/L_vs_CD, the Gap statistic) of *S. sesuvioides*. **(S)** Transcripts up-regulated in leaves, as compared to cotyledons night (Up_L/L_vs_CN, the Gap statistic) of *S. sesuvioides*. **(T)** Transcripts up-regulated in cotyledons, as compared to leaves of *S. sesuvioides* (Up_CD/L_vs_CD, the Gap statistic) **(U)** Transcripts up-regulated in cotyledons night, as compared to leaves (Up_CN/L_vs_CN, the Gap statistic) of *S. sesuvioides*.
