# Supplementary material for: C4-like Sesuvium sesuvioides (Aizoaceae) exhibits CAM in cotyledons and putative C4-like + CAM metabolism in adult leaves as revealed by transcriptome analysis
Source: BMC Genomics. 2024 Jul 13;25:688. doi: 10.1186/s12864-024-10553-2 (PMC11245778; doi:10.1186/s12864-024-10553-2)
Supplement: Supplementary file 7 — Additional file 7: Fig. S5. K-means clustering of DE transcripts between leaves and cotyledons of S. seuvioides and between C3 and C4 species. [file 12864_2024_10553_MOESM7_ESM.pdf]

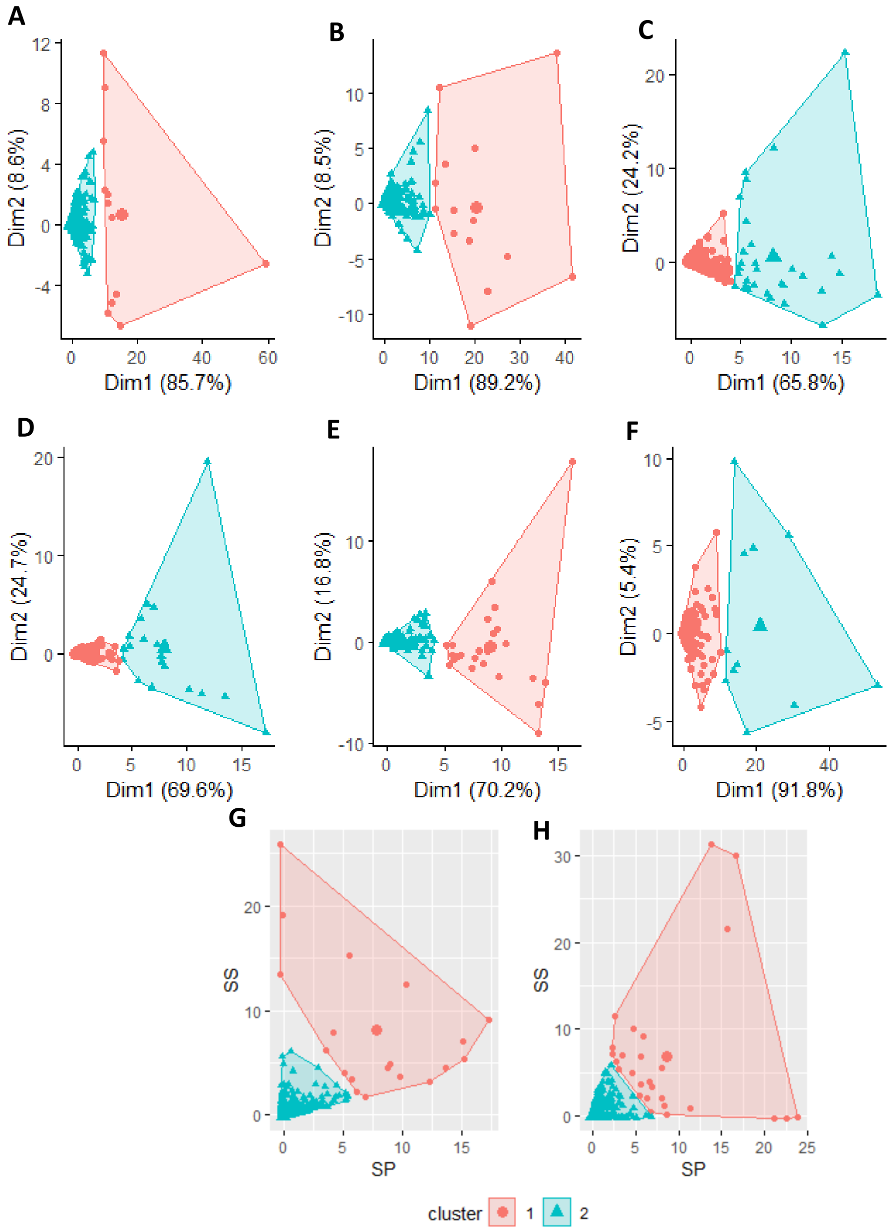

Additional file 7: Fig. S5. K-means clustering of differentially expressed transcripts between leaves and cotyledons of S. seuvioides and between C_3 and C_4 species. **(A)** Up-regulated transcripts in leaves compared to CD (Up-L/L-vs-CD), **(B)** Up-regulated transcripts in CD compared to leaves (Up-CD/L-vs-CD), **(C)** Up-regulated transcripts in CN compared to CD (Up-CN/CN-vs-CD), **(D)** Up-regulated transcripts in CD compared to CN (Up-CD/CN-vs-CD), **(E)** Up-regulated transcripts in L compared to CN (Up-L/L-vs-CN), **(F)** Up-regulated transcripts in CN compared to L (Up-CN/L-vs-CN), **(G)** Up-regulated transcripts in C_4 compared to C_3 (Up-C_4/C_4-vs-C_3), **(H)** Up-regulated transcripts in C_3 compared to C_4 (Up-C_3/C_4-vs-C_3). Colours represent clusters
